# Novel Human Podocyte Cell Model Carrying G2/G2 APOL1 High-Risk Genotype

**DOI:** 10.3390/cells10081914

**Published:** 2021-07-28

**Authors:** Pepe M. Ekulu, Oyindamola C. Adebayo, Jean-Paul Decuypere, Linda Bellucci, Mohamed A. Elmonem, Agathe B. Nkoy, Djalila Mekahli, Benedetta Bussolati, Lambertus P. van den Heuvel, Fanny O. Arcolino, Elena N. Levtchenko

**Affiliations:** 1 Department of Development and Regeneration, Katholieke Universiteit Leuven, 3000 Leuven, Belgium; drmfutu@yahoo.fr (P.M.E.); christiana.adebayo@kuleuven.be (O.C.A.); jeanpaul.decuypere@kuleuven.be (J.-P.D.); agathe.nkoybikupe@student.kuleuven.be (A.B.N.); djalila.mekahli@uzleuven.be (D.M.); bert.vandenheuvel@kuleuven.be (L.P.v.d.H.); elena.levtchenko@uzleuven.be (E.N.L.); 2 Department of Paediatrics, Division of Nephrology, Faculty of Medicine, University Hospital of Kinshasa, University of Kinshasa, Kinshasa, Democratic Republic of the Congo; 3 Centre for Molecular and Vascular Biology, Department of Cardiovascular Sciences, Katholieke Universiteit Leuven, 3000 Leuven, Belgium; 4 Department of Molecular Biotechnology and Health Sciences, University of Turin, 10124 Turin, Italy; linda.bellucci@unito.it (L.B.); benedetta.bussolati@unito.it (B.B.); 5 Department of Clinical and Chemical Pathology, Faculty of Medicine, Cairo University, Cairo 11628, Egypt; mohamed.abdelmonem@kasralainy.edu.eg; 6 Department of Paediatrics, Division of Nephrology, University Hospitals Leuven, 3000 Leuven, Belgium; 7 Department of Paediatric Nephrology, Radboud University Medical Centre, 6500 Nijmegen, The Netherlands

**Keywords:** Apolipoprotein L1, podocyte, kidney dysfunction, kidney disease cellular model, urine-derived cells

## Abstract

Apolipoprotein L1 (*APOL1*) high-risk genotypes (HRG), G1 and G2, increase the risk of various non-diabetic kidney diseases in the African population. To date, the precise mechanisms by which *APOL1* risk variants induce injury on podocytes and other kidney cells remain unclear. Trying to unravel these mechanisms, most studies have used animal or cell models created by gene editing. We developed and characterised conditionally immortalised human podocyte cell lines derived from urine of a donor carrying *APOL1* HRG G2/G2. Following induction of APOL1 expression by polyinosinic-polycytidylic acid (poly(I:C)), we assessed functional features of APOL1-induced podocyte dysfunction. As control, APOL1 wild type (G0/G0) podocyte cell line previously generated from a Caucasian donor was used. Upon exposure to poly(I:C), G2/G2 and G0/G0 podocytes upregulated APOL1 expression resulting in podocytes detachment, decreased cells viability and increased apoptosis rate in a genotype-independent manner. Nevertheless, G2/G2 podocyte cell lines exhibited altered features, including upregulation of CD2AP, alteration of cytoskeleton, reduction of autophagic flux and increased permeability in an in vitro model under continuous perfusion. The human APOL1 G2/G2 podocyte cell model is a useful tool for unravelling the mechanisms of APOL1-induced podocyte injury and the cellular functions of APOL1.

## 1. Introduction

Individuals with African ancestry have been reported to develop kidney failure at a higher rate than other races [1]. A portion of this racial disparity has been explained by variants in the Apolipoprotein L1 (*APOL1*) gene [2]. Inheriting two copies of the *APOL1* risk variants (RVs), termed G1 and G2, have been reported to significantly increase the risk of various kidney diseases [3,4,5,6,7].

Prior to establishing its role in kidney diseases, *APOL1* was known to protect against certain trypanosome species in humans and some primates [7]. APOL1 circulates in blood as part of a high-density lipoprotein (HDL) complex that also contains apolipoprotein A1 (APOA1) and haptoglobin related protein (HPR). This complex named trypanosome lytic factor (TLF) confers innate protection against subspecies of *Trypanosoma brucei* (T.b.) [8,9]. *T.b. rhodesiense* and *T.b. gambiense* have evolved a mechanism to evade the TLF lysis. *T.b. rhodesiense* evades lysis by expressing serum resistance associated (SRA) protein that binds to the C-terminus domain of the APOL1, in the process neutralising its lytic activity leading to infection of the host and the development of African sleeping sickness [10,11,12,13,14]. In turn, *T.b. gambiense* resists TLF via multifactorial defence mechanisms [14,15]. *APOL1* risk variants G1 and G2 restore the lytic activity of human serum, providing selective advantage to carriers against African sleeping sickness caused by *T.b. rhodesiense,* but not *T.b.* *gambiense* [16].

APOL1 is expressed in various tissues such as lung, placenta, pancreas, liver and kidney. In the kidney, different cell types express APOL1, including extra glomerular vascular endothelial cells, podocytes and proximal tubular cells [9,17]. However, to date, reported data on kidney disease associated with *APOL1* RVs have been mostly limited to podocytes [18,19].

Most individuals carrying two *APOL1* RVs do not develop kidney disease; this suggests that additional cellular stressors such as inflammation are required to upregulate APOL1 and induce podocyte injury [18,20]. Therefore, a better understanding of APOL1-induced podocyte injury is still needed in order to derive effective therapies [18]. As such, cell and animal models overexpressing APOL1 G1 and G2 variants showed increased cytotoxicity leading to apoptosis, necrosis or inflammatory cell death (pyroptosis) [21,22,23,24], disrupted autophagic flux [18], altered mitochondrial function [25], increased lipid accumulation [26], increased potassium efflux and enhanced stress response pathways [27]. In most of these studies, a synthetic double-stranded RNA, polyinosinic-polycytidylic acid (poly(I:C)), which is an agonist of Toll-like receptor 3 (TLR3), was used for the activation of APOL1 driven by upregulation of interferon. 

In other in vitro cell models, APOL1 overexpression was generated using gene editing techniques such as viral vector transductions or CRISPR-Cas9 technology [22,23,27]. Due to the artificial modifications, it is uncertain whether these models fully reflect the actual cellular phenotype of the patients [5]. In our study, we generated a podocyte cell model derived directly from a human carrying *APOL1* high-risk genotype (HRG), G2/G2. Using this model, we demonstrate pathologic features of APOL1 G2/G2 podocytes in comparison with a reference podocyte cell line, carrying *APOL1* allele G0/G0.

## 2. Materials and Methods

### 2.1. Ethics

This study (study number S61246; registration number B322201838275) was approved by the Ethical Committee of the University Hospital Leuven (Ethische Commissie Onderzoek UZ/KU Leuven on 18 July 2018 and carried out in accordance with the guidelines. The participants were recruited from the African community living in Belgium after signing an informed consent form.

All methods in this study were performed in accordance with relevant institutional guidelines and regulations.

### 2.2. Reagents and Antibodies

All reagents were of analytical grade or used as specified. The antibodies used in this study include anti-synaptopodin (65194, Progen Biotechnik, Heidelberg, Germany), anti-podocin (EB12149, Everest Biotech Ltd., Oxfordshire, UK), anti-paxillin (610569, BD Biosciences, Hackensack, NJ, USA), anti-podocalyxin (ab154305, abcam, Cambridge, United Kingdom), anti-LC3-II (0231-100/LC3-5F10, Nanotools, Teningen, Germany), anti-β-Actin (#4970, Cell Signalling Technology, Leiden, The Netherlands), polyclonal Goat Anti-mouse IgG-HRP (P044701-2, DAKO, Santa Clara, CA, USA) and polyclonal Goat Anti-rabbit IgG-HRP (P044801-2, DAKO, Santa Clara, CA, USA). Alexa Fluor-488-conjugated phalloidin was obtained from Invitrogen (a12379, Waltham, MA, USA), as well as Alexa-546-conjugated secondary antibodies. Poly (I:C) (TLRL-PIC) was purchased from Invivogen (San Diego, CA, USA) and used in a concentration of 50 µg/mL. Radioimmunoprecipitation assay (RIPA) buffer containing 10 mM sodium phosphate, 150 mM NaCl, 1.5 mM MgCl_2_, 0.5 mM Dithiothreitol (DTT) and 1% Triton X-100, complete EDTA-free protease inhibitor tablets (Sigma-Aldrich, St. Louis, MO, USA) were used to prepare protein lysate. Fluorescein isothiocyanate (FITC)-Annexin V/Propidium Iodide (PI) for flow cytometry (V13242, Invitrogen, Waltham, MA, USA) were used for apoptosis assay. WST-1 reagent (11644807001, Sigma-Aldrich, St. Louis, MO, USA) was used for cytotoxicity assay.

### 2.3. Participants and APOL1 Genotyping 

Since the podocyte isolation requires fresh urine, participants were recruited among African community living in Belgium (*n* = 89). From the whole blood samples collected, DNA was extracted using QIAGEN kit following the manufacturer’s instructions (QIAamp^®^ DNA Mini kit; QIAGEN, Venlo, The Netherlands) in the laboratory of Paediatric Nephrology, Katholieke Universiteit (KU) Leuven, Belgium. *APOL1* genotyping was performed for two kidney risk alleles (G1 and G2) as previously described [5]. *APOL1* high-risk genotype (HRG) was defined by the presence of two risk variants (G1/G1, G2/G2 or G1/G2) while low-risk genotype (LRG) was defined by the presence of zero or one risk variant.

Blood and urine samples were collected from those who were identified as carrying HRG to assess kidney function via estimated glomerular filtration rate (eGFR) and urinary protein excretion. Serum creatinine and random spot urine protein/creatinine (P/C) ratio were measured according to standard laboratory protocols. eGFR was calculated using the Schwartz formula [28] for participants aged less than 18 years and the Cockcroft–Gault (C-G) equation [29] for those aged above 18 years. 

### 2.4. Isolation and Culture of Podocytes Exfoliated into Urine

From the participants in whom *APOL1* HRG has been detected (6 out of 89), freshly voided urine was collected, and exfoliated cells were cultured as previously described [30]. In brief, urine was centrifuged at 200 g for 5 min, the pellet was washed in PBS and re-suspended in DMEM-HAM’s F-12 (Lonza, Basel, Switzerland) with 10% foetal bovine serum (Gibco, Waltham, MA, USA), 50 IU/mL penicillin and 50 mg/mL streptomycin (Lonza, Basel, Switzerland), 5 µg/mL insulin, 5 µg/mL transferrin and 5 ng/mL selenium (Sigma-Aldrich, St. Louis, MO, USA) (podocyte medium). Cells were immortalised and sub-cloned using a temperature-sensitive SV40-hTERT viral system. Cells grew at a permissive temperature of 33 °C and, prior to the experiments, they were incubated at 37 °C for 10 days to ensure growth arrest and differentiation. APOL1 WT (G0/G0) podocytes used as control were already available and previously generated from a Caucasian donor [31]. 

### 2.5. Quantitative Polymerase Chain Reaction (qPCR)

The expression of the specific podocyte markers synaptopodin, podocalyxin and Cluster of Differentiation 2-Associated Protein (*CD2AP*) was analysed by qPCR in the clonal cell lines isolated from G2/G2 carrier and compared with APOL1 G0/G0 podocytes. qPCR was also used to analyse the expression of *APOL1* in cell clones before and after 24 h of incubation with Poly I:C (50 µg/mL). Beta-actin was used as reference gene (Table 1). RNA was isolated using RNeasy Micro Kit (Qiagen GmbH, Hilden, Germany) according to the manufacturer’s instructions. A total of 500 ng of RNA was used to synthesise cDNA using a mix of Oligo (dT) 12–18 Primer (Invitrogen: 18418012; Waltham, Massachusetts, USA), random hexamer primers (Invitrogen: 48190011; Waltham, Massachusetts, USA), dNTP mix (100 mM Invitrogen: AM8228G; Waltham, Massachusetts, USA) and SuperScript^®^ III reverse transcriptase, plus 5× first-strand reaction mix (Invitrogen: 18080085; Waltham, MA, USA). qPCRs were performed in triplicate using StepOnePlus™ real-time PCR system (Thermo Fisher Scientific; Waltham, MA, USA) with Platinum SyberGreen qPCR Supermix (Invitrogen: 11744-500; Waltham, Massachusetts, USA), 10 µM of primers and 1.5 µL of cDNA. Results were analysed using StepOnePlus™ software. 

### 2.6. Immunofluorescence Staining

The immunofluorescence staining was performed for the podocyte specific proteins synaptopodin (65194, Progen Biotechnik), podocalyxin (ab154305, abcam) and podocin (EB12149, Everest Biotech), as well as for analysing the podocyte cytoskeleton (phalloidin staining; A12370, Life Technologies) and adhesion site (paxillin staining; 610569, BD Biosciences). Briefly, cells grown on glass coverslips were fixed with 4% paraformaldehyde for 10 min at room temperature and washed once in PBS. They were permeabilised with 0.1% Triton X-100 (Sigma-Aldrich, St. Louis, MO, USA) for 5 min on ice and washed twice with PBS. Blocking solution (0.5% BSA, 0.2% gelatin, 0.5% FBS in PBS) was added to cells for 1 h. Cells were incubated with primary antibodies overnight at 4 °C and further with AlexaFluor secondary antibodies for 1 h in dark at room temperature. 4′, 6-diamidino-2-phenylindole (DAPI) was diluted to 1:1000 in mounting medium (S302380-2, DAKO, Santa Clara, CA, USA) for nuclear staining. The cells were washed 5 times with PBS between steps. Samples were examined with a fluorescence microscope (Olympus BX41, Olympus Belgium NV, Antwerpen, Belgium). Image processing and analysis were performed using Image J software. 

### 2.7. Detachment Assay

To analyse detachment of cells in culture upon upregulation of APOL1 induced by poly(I:C), 20,000 APOL1 HRG podocytes and control podocytes were seeded in a 6-well plate. After 7 days at 37 °C, cells were incubated with poly(I:C) (50 µg/mL) for 24 h. The supernatant was collected, and detached cells were counted using the TC20™ Automated Cell Counter (Bio-Rad Laboratories, Temse, Belgium), which estimates the number of live and dead cells in the cells’ suspension. Percentage of detachment was represented as (number of detached cells)/(number of detached + attached cells). Three independent experiments were performed with three technical replicates in each.

### 2.8. Cytotoxicity Assay 

APOL1-mediated cytotoxicity in G2/G2 podocyte cell lines and in control cells was measured using a water-soluble tetrazolium salt (WST-1) cytotoxicity assay (11644807001, Sigma-Aldrich, St. Louis, MO, USA) according to manufacturer instructions. WST-1 is reduced in metabolically active cells to produce a blue precipitate, formazan. The detection of formazan level in the cells allows quantifying the number of viable cells. Briefly, cells were grown in culture, trypsinised and seeded at a density of 20,000 cells per well in a 96-well plate. Cells were incubated overnight in 33 °C to allow attachment, and then transferred to 37 °C for 10 days. Poly(I:C) (50 µg/mL) was added for APOL1 induction. After at least 16 h of incubation, 10 µL of WST-1 was added in 100 µL of medium per well and cells were incubated further for 2 h at 37 °C. Then, absorbance was measured at 450 nm versus a 620 nm reference by using an ELISA reader. Three independent experiments were performed with three technical replicates in each. 

### 2.9. FITC-Annexin V/PI Staining for Apoptosis Assay 

To analyse the APOL1-induced apoptotic cell death pathway, human HRG podocyte cell lines and controls treated or not with poly(I:C) were stained using the FITC-AnnexinV apoptosis detection kit and PI kit according to the manufacturer’s instructions. Samples were analysed by fluorescence-activated cell sorting (FACS) within 30 min. The analysis allowed obtaining the proportion of live, early apoptotic, late apoptotic and necrotic cells contained in each sample. Two independent experiments were performed.

### 2.10. Western Blot for Autophagy Analysis 

Western blot was used to investigate the expression and possible modifications of LC3-II involved in autophagy. Immunodetection of LC3-II (0231-100/LC3-5F10, mouse monoclonal; NanoTools, Teningen, Germany) was performed in basal conditions and after 24 h of incubation with poly(I:C) in G0/G0 and G2/G2 podocytes. However, detection of LC3-II only shows a snapshot of protein levels in the cell without reproducing the dynamic information of the autophagic process, so-called autophagic flux. As autophagy is a continuously ongoing process, an increased level of LC3-II may result from either increased production or a decreased degradation of autophagosomes. To assess autophagic flux, cells were treated with 100 nM Bafilomycin A1 (Baf A1) (LC Laboratories, Woburn, MA, USA) or dimethylsulfoxide (DMSO) (Sigma-Aldrich, St. Louis, MO, USA) as vehicle for 3 h before collecting pellet, as previously described [32]. Baf A1 is a lysosomal inhibitor and autophagic flux blocker. It blocks the fusion of autophagosomes and lysosomes leading to an accumulation of the autophagosomal marker LC3-II. As such, in the presence of Baf A1, information is gathered only about LC3-II (and autophagosome) production. For the blotting, cells were lysed with 50–100 µL of RIPA buffer. The protein concentration was determined using BCA protein assay kit (ThermoFisher Scientific, Waltham, MA, USA). Equivalent amounts of protein were separated on a NuPAGE^TM^ 4–12% Bis-Tris Protein Gel (Invitrogen, Waltham, MA, USA) and followed by blotting on polyvinylidene difluoride (PVDF) membrane. Primary and secondary antibodies were used according to the manufacturer’s instructions. Blots were developed using HRP-conjugated secondary antibodies and analysed using Image J software. Three independent experiments were performed. Full-length blots are presented in Supplementary Figure S1.

### 2.11. Perfusion Assay for Glomerular Membrane Permeability Assessment Using G2/G2 Podocytes 

To perform the filtration assay, a dynamic millifluidic culture system (IVtech Srl, Lucca, Italy) was used. A total of 80,000 differentiated podocytes were seeded on PET porous membrane (ipCELLCULTURE^TM^ Track Etched Membrane, it4ip S.A., Louvain-la-Neuve, Belgium) embedded in the central part of a small (2.5 mL of volume capacity) bioreactor. The membrane divides the cell-chamber into 2 compartments, each with an inlet and outlet channel, to allow, once connected the circuit, the continuous flow of fluid in one direction only. In case of administration of poly(I:C), the day after the setup of culture in the bioreactor, podocyte-medium were changed with medium plus 50 μg/mL poly I:C, otherwise a normal change in medium was carried out. After 16 h of poly(I:C) incubation, or 2 days after setting the culture, the perfusion assay was performed. Firstly, in an incubator, the bioreactor was connected to the main body of the system, capable of delivering liquid at a certain flow rate (in this case, 100 μL/min), directly into the bioreactor. During the filtration assay, in each circuit, a peristaltic pump allows the entry of medium with 1 mg/mL Albumin–fluorescein isothiocyanate conjugate (Sigma-Aldrich, St. Louis, MO, USA) in the lower part of the bioreactor and in the upper part instead of normal medium. After 3 h of perfusion, the liquid coming out of the two compartments of the bioreactor were collected separately and fluorescence was measured in triplicate. A higher fluorescence indicates lower podocyte integrity.

### 2.12. Statistical Analysis

Statistical analysis was performed using Microsoft Excel^®^ and GraphPad Prism^®^ software. Data from at least three independent experiments were analysed for each condition, unless otherwise stated in methods. Immunofluorescence images were obtained from randomly selected cells from three independent experiments, and the images shown are representative of the majority of cells. Statistical significance was evaluated using Student’s *t*-test for detecting differences of two groups and one-way analysis of variance (ANOVA) for detecting differences between more than two groups. Analysis was considered to be statistically significant when *p* < 0.05 (* *p* < 0.05; ** *p* < 0.01; *** *p* < 0.001; **** *p* < 0.0001).

## 3. Results

### 3.1. Generation of a Novel Podocyte Model from a Human Carrying APOL1 High-Risk Genotype 

#### 3.1.1. APOL1 Genotyping and Culture of Cells Harvested from APOL1 HRG Carriers

The study population was recruited among the African community living in Belgium and *APOL1* sequencing analysis was performed. Out of 89 recruited participants, six (6.7%) carried HRG, being G1/G1 (*n* = 1), G2/G2 (*n* = 1) and G1/G2 (*n* = 4) (Table 2). The low-risk genotype (LRG) frequencies were 55.0%, 23.5% and 14.6% for G0/G0, G1/G0 and G2/G0, respectively. After collection of urine samples from the six participants carrying *APOL1* HRG, only cells harvested from one G2/G2 carrier who had stage 3 chronic kidney disease and proteinuria proliferated successfully and twelve conditionally immortalised clones were generated.

#### 3.1.2. Characterisation of APOL1 G2/G2 Podocyte Cell Model 

**Figure 1:**
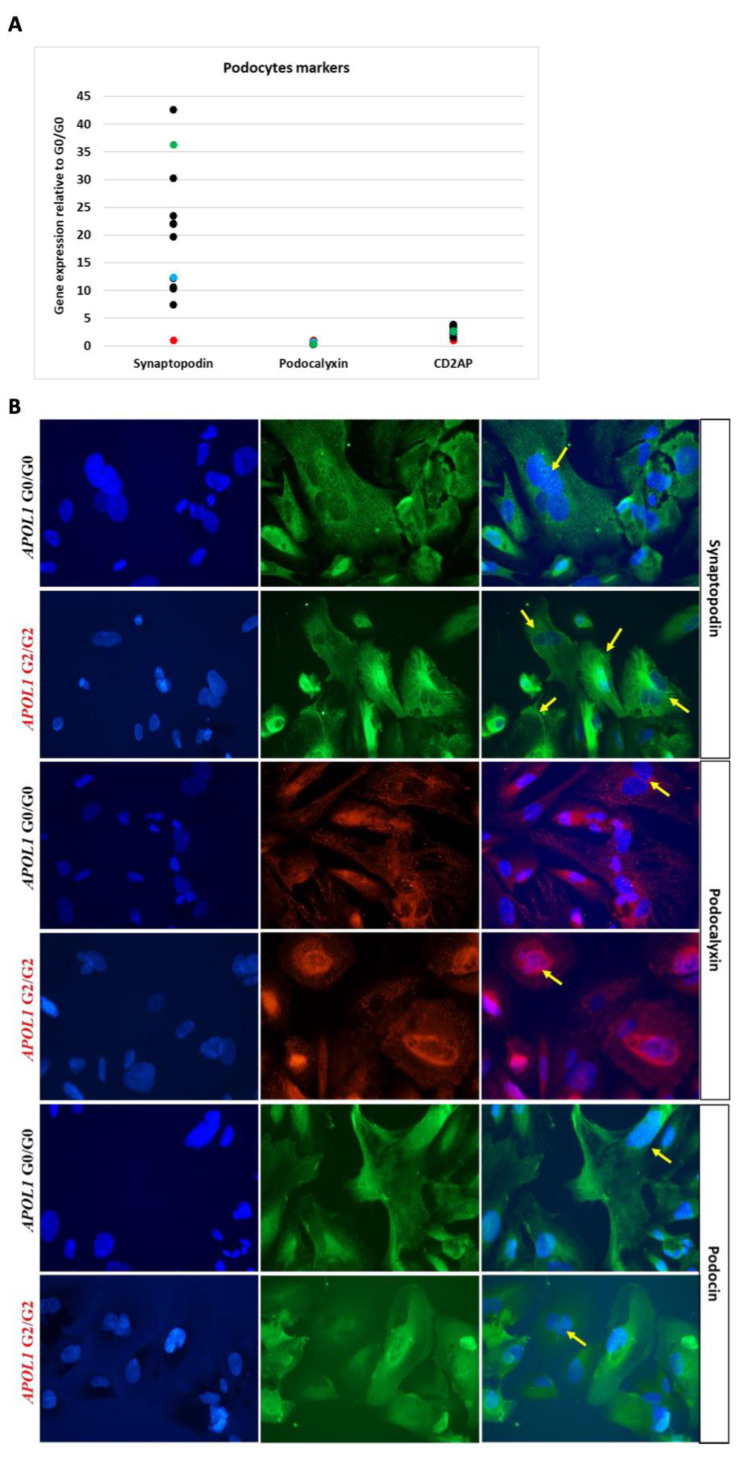
Characterisation of human podocytes obtained from urine of the donor carrying APOL1 G2/G2. (**A**) Quantitative polymerase chain reaction (qPCR) analysis of expression of podocyte-specific genes synaptopodin; CD2-associated protein gene (CD2AP) and podocalyxin in twelve cell clones of APOL1 G2/G2 podocytes normalised to G0/G0 expression. Each dot represents mean ± SD of each clone; red dot is the control APOL1 G0/G0, blue dot is APOL1 G2/G2 clone 1 and green dot is APOL1 G2/G2 clone 2, selected as representative for further experiments. (**B**) Immunofluorescence staining of cultured podocytes for podocyte-specific proteins synaptopodin (green), podocin (green), podocalyxin (red) and the nuclear marker 4′,6-diamidino-2-phenylindole (DAPI) (blue); arrows point to bi- or multi-nucleated cells representing characteristic podocyte feature. Magnification: 400×.

We amplified and sequenced the exon 7 (883 bp) of *APOL1* for the variant G2 from gDNA isolated from the APOL1 G2/G2 clones and a podocyte control cell line (APOL1 G0/G0). Then, we evaluated the expression of specific podocyte genes synaptopodin, podocalyxin and CD2AP in twelve clones isolated from APOL1 G2/G2 carrier in comparison with APOL1 G0/G0 podocytes (Figure 1A). All tested podocyte genes were expressed in APOL1 G2/G2 podocytes as well as the podocyte-specific proteins synaptopodin, podocalyxin and podocin (Figure 1B). Interestingly, all G2/G2 podocyte clones expressed significantly higher levels of synaptopodin in comparison with G0/G0 control (red dot, Figure 1A). Based on these data, we chose two representative clones to further assess functionality (clone 1 blue dot and clone 2 green dot in Figure 1A). 

### 3.2. Functional Features of Podocyte Dysfunction in Human APOL1 G2/G2 Podocyte Model

**Figure 2:**
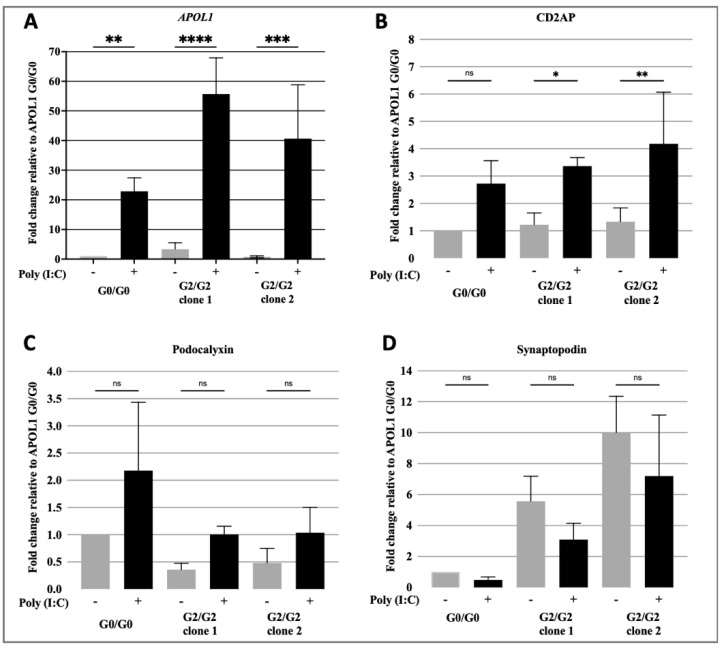
Changes in gene expression upon APOL1-induction. qPCR analysis of expression of the APOL1 (**A**) and podocyte-specific genes CD2-associated protein (CD2AP) (**B**); podocalyxin (**C**) and synaptopodin (**D**) in APOL1 G0/G0, APOL1 G2/G2 clones 1 and 2 following incubation with poly (I:C) for 24 h. Each bar represents the mean ± SD of three independent experiments, performed in technical triplicates. * *p* < 0.05; ** *p* < 0.01; *** *p* < 0.001; **** *p* < 0.0001.

Overexpression of APOL1-G1 and G2 variants has been proposed as a mechanism of APOL1-associated kidney diseases mainly affecting podocytes [18,24,26]. We performed various cell biological assays and assessed podocyte specific functions to validate our cell lines as a model to study mechanisms of APOL1-associated kidney disease. Overexpression of APOL1 was induced by incubation of podocytes with poly(I:C) for 24 h. Results showed that poly(I:C) induced similar levels of APOL1 overexpression in both G0/G0 and G2/G2 podocytes (Figure 2A). 

We then tested whether poly(I:C) treatment influenced expression of podocyte-specific genes and observed a significant upregulation of CD2AP (Figure 2B). There was also a trend in upregulation of podocalyxin and downregulation of synaptopodin, but these changes in expression were not statistically significant (Figure 2C,D).

#### 3.2.1. APOL1-Induced Cytotoxicity 

**Figure 3:**
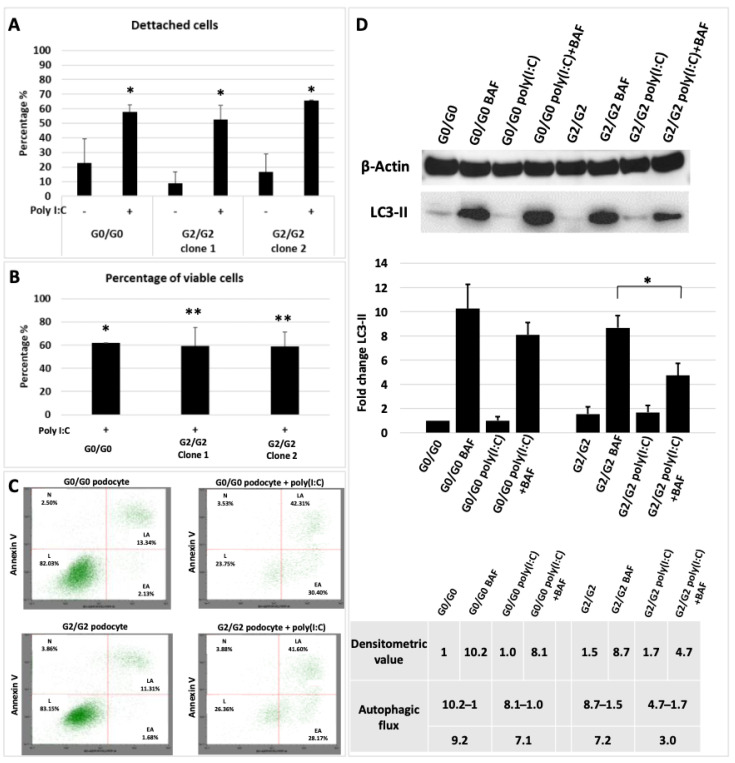
APOL1-induced cell toxicity. (**A**) Proportion of detached APOL1 G0/G0 and APOL1 G2/G2 podocytes by the total number of cells before and after 24 h of incubation with poly(I:C). Each bar represents mean ± SD of three independent experiments, performed in technical triplicates. * *p* < 0.05. (**B**) The WST-1 assay in two clones of G2/G2 and one G0/G0 podocyte cell lines following incubation with poly(I:C) (50 µg/mL) demonstrated similar cytotoxicity upon APOL1 upregulation in all cell lines. Each bar represents mean ± SD of three independent experiments with three technical replicates for each cell line. * *p* < 0.05; ** *p* < 0.01 (**C**) APOL1 G2/G2 podocyte with or without poly(I:C) (lower panels) and control cells (upper panels) were analysed by flow cytometry after FITC-annexinV/PI staining. The spot plots show the percentage of cells in each quadrant (L, live; EA, early apoptotic; LA, late apoptotic; N, necrotic) from a representative replicate of two independent experiments. (**D**) LC3-II Western-blot analysis of G0/G0 and G2/G2 podocyte cell lines: basal level (G0/G0; G2/G2); after treatment with bafilomycin A (Baf, 100 nM) to induce the autophagic block; after 24 h incubation with poly(I:C) (50 ng/mL) to induce upregulation of APOL1; and combined induction with poly(I:C) and treatment with bafilomycin A (poly I:C+Baf). Full-length blots are presented in Supplementary Figure S1. Fold-change quantification of the protein abundance of LC3-II relative to β-actin after immunoblotting in each cell line condition normalised to G0/G0 baseline. Assessment of autophagic flux using densitometric analysis of the LC3-II band (table).

*Cell detachment:* Normal podocyte density (number of cells per glomerular tuft volume) is necessary to maintain the integrity of glomerular filtration barrier. Reduction in podocyte density, caused by increased glomerular volume and/or reduced podocyte number is associated with proteinuria, glomerulosclerosis and kidney failure in various progressive glomerular diseases [33]. It has been recently proposed in a mouse model of HIV-associated nephropathy that APOL1-G0 expression in podocytes has a protective function against podocyte loss or injury when exposed to an environmental stressor. This protective function was absent in mice carrying APOL1-G2 variant [34]. We analysed podocyte detachment in culture upon overexpression of APOL1 induced by poly(I:C) in APOL1 G2/G2 versus APOL1 G0/G0 cell lines. The results showed a high percentage of detached cells, but no significant difference in podocyte detachment related to the APOL1 genotype (Figure 3A). 

*Cell viability:* Upregulation of APOL1 in G0, G1 and G2 variants was shown to cause cell death in mammalian cell lines or model organisms [21,22,23,35]. Therefore, we investigated whether our human APOL1 G2/G2 podocytes were less viable upon APOL1 upregulation. The results showed that indeed the proportion of viable cells was significantly reduced in APOL1 G2/G2 podocyte clones (from 100% to 59.17% ± 12.2% in clone 1 and to 59.01% ± 11.9% in clone 2) as well as in the control APOL1 G0/G0 podocytes (from 100% to 61.93% ± 16.2%) (Figure 3B). Nevertheless, this reduction in cell viability was not dependent on the *APOL1* genotypes. 

*Cell death:* Enhanced cell death rate has been reported among various pathways leading to kidney damage associated with APOL1 RVs [27]. To evaluate cell death effect driven by APOL1 overexpression in the human G2/G2 podocyte model, we analysed apoptosis and necrosis by flow cytometry before and after incubation with poly(I:C). The results showed increased apoptosis rather than necrosis in APOL1 podocyte cell lines regardless of the genotype, suggesting that increased apoptosis owing to APOL1 induction is variant-independent (Figure 3C).

*Autophagy:* Additionally, APOL1 overexpression has been associated with altered autophagic cell death mechanisms [36]. Therefore, we evaluated the effect of APOL1 upregulation on autophagy in APOL1 G2/G2 podocyte clones and in APOL1 G0/G0 podocytes as control. Autophagy is a dynamic process, involving the generation as well as the degradation of autophagosomes. Accumulation of LC3-II, a marker for the number of autophagosomes indicates either the induction of autophagy or the inhibition of lysosomal function and/or fusion of autophagosomes with lysosomes [37]. Therefore, the determination of autophagic flux (turnover of LC3-II) is essential to assess and differentiate between the induction and suppression of autophagy. Blockage of autophagic degradation by lysosomal vacuolar-type H+-ATPase inhibitor Bafilomycin (Baf) A1 allowed the evaluation of autophagic flux in podocytes. After exposure to poly(I:C) in the presence of Baf A1, LC3-II accumulation was lower in both APOL1-G0 and G2 podocytes. However, the expression was significantly reduced in G2/G2 podocytes compared to G0/G0 (Figure 3D), indicating reduced generation of autophagosomes. Densitometric analysis suggested an overall reduction in autophagic flux of APOL1 G2/G2 podocytes compared with G0/G0 (9.2 vs. 7.1 without and with Baf A1, respectively, in G0/G0 and 7.2 vs. 3.0 in G2/G2) (Figure 3D, table). 

#### 3.2.2. APOL1-Induced Alteration of Cytoskeleton and Filtration Barrier

**Figure 4:**
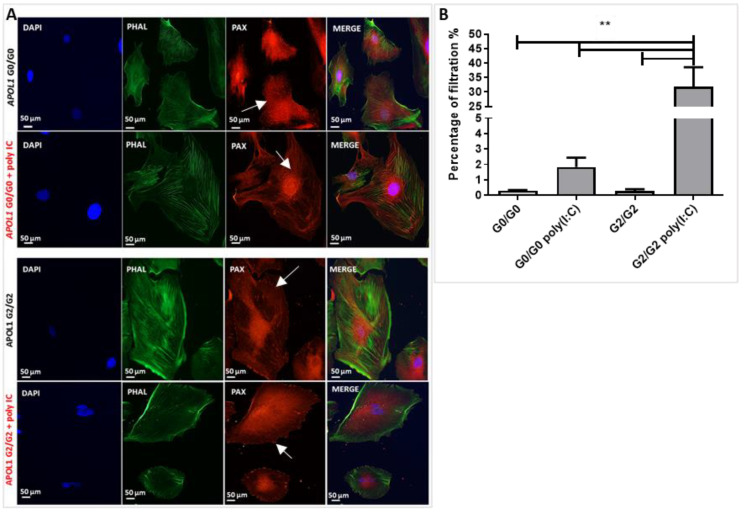
APOL1-induced cytoskeleton abnormalities. (**A**) Staining for actin filaments using phalloidin (phal) (green) and focal adhesion molecules using paxillin (pax) (red dots; arrows) and nuclear DAPI (blue) in APOL1 G0/G0 control cells and in APOL1 G2/G2 podocytes at basal conditions and after APOL1 induction by poly(I:C). (**B**) After 48 h of podocyte/GEC co-culture, the passage of FITC-BSA from the lower to the upper compartment was measured in the dynamic system during 3 h. APOL1 G2/G2 and G0/G0 podocyte cell lines showed similar permeability, which was significantly increased by poly(I:C) treatment only in the G2/G2 podocytes. Each bar represents mean ± SD of three independent experiments with three technical replicates for each cell line. ** *p* < 0.01.

Podocytes exhibit a unique cytoskeletal architecture, which is linked to their function in maintaining the kidney filtration barrier [38]. Normally, loss of F-actin stress fibres and changes in lamellipodia mimic podocyte foot process effacement in vivo. We analysed podocyte cytoskeleton and distribution of adhesion sites in G2/G2 podocytes compared with G0/G0 in basal conditions and after exposure to poly(I:C). Actin filaments were significantly reduced and disorganised in G2/G2 podocytes in comparison with G0/G0 podocytes. Furthermore, after APOL1 induction, G2/G2 podocytes exhibited fewer adhesion sites than observed in basal conditions and when compared with G0/G0 podocytes (Figure 4A).

Impairment of the podocyte cytoskeletal apparatus by genetic mutations results in proteinuria, dysfunctional glomerular filtration and consequent glomerular disease [38]. We used a permeability assay in dynamic condition to model in vitro the glomerular permeability, adopting a millifluidic system that allows the continuous perfusion in co-culture under 8 × 10^−5^ dyn/cm^2^ shear stress, as previously described [39]. Permeability was assessed by the evaluation of the passage of albumin across a barrier formed by APOL1 G2/G2 podocytes and glomerular endothelial cell (GEC) co-cultures, before and after incubation with poly(I:C) for 24 h, during constant flux. In fact, our results showed that the in vitro glomerular barrier formed by APOL1 G2/G2 podocytes and endothelial cells treated with poly(I:C) exhibited significantly higher permeability to albumin compared to podocytes at basal conditions (Figure 4B). 

Taken together, these data indicate that human APOL1 G2/G2 podocyte model can mirror in vitro the renal cytopathology observed in patients carrying *APOL1* HRG, representing a novel tool to support the investigation of APOL1-related kidney diseases and to test new therapies.

## 4. Discussion

The strong association between *APOL1* gene expression with several non-diabetic chronic kidney diseases and its mechanisms have been extensively investigated using animal or cell models created by gene editing [22,23,27]. Our group has an established methodology to generate stable kidney epithelial lines from cells exfoliated into urine of healthy subjects and patients [30,40,41,42,43]. Using the same approach, we successfully generated APOL1 G2/G2 podocyte cell model from urine of a human donor carrying *APOL1* HRG (G2/G2). The obtained podocyte cell lines were characterised by showing expression of podocyte genes and proteins and by demonstrating functional features of APOL1-induced injury when treated with poly(I:C). To our knowledge, this is the first G2/G2 human podocyte cell model derived from a patient carrying this genotype and it can be of great interest for researchers investigating cellular functions and mechanisms of APOL1.

Using the new G2/G2 cell model, we confirmed several cellular phenotypes demonstrated in previous studies, indicating the auxiliary value of our model for unravelling molecular pathways of APOL1-related kidney cell damage. In fact, our G2/G2 podocyte cell model strongly supported the findings of Uzureau et al. [44], who recently showed that the disease phenotype linked to the expression of C-terminal APOL1 risk variants is related to the reduction in phosphatidylinositol-4-phosphate (PI(4)P) synthesis at the Golgi by preventing APOL3 from activating the Golgi PI(4)-kinase IIIB (PI4KB).

The endogenous expression of APOL1 is induced under inflammatory conditions, such as viral infections, primarily activating the Toll-like receptor 3 (TLR3) via increased type 1 interferons, which is mimicked in vitro by incubation of cells with poly(I:C) [21,44]. Indeed, our G2/G2 and G0/G0 podocytes upregulated APOL1 expression upon incubation with poly(I:C), regardless of the *APOL1* genotype. Subsequently, we examined the risk variant-dependent cytotoxicity induced by the upregulation of APOL1 in G2/G2 podocytes compared with control G0/G0 cells. We found that APOL1 overexpression induced podocytes detachment, decreased cells viability and increased apoptosis rate in both genotypes at similar levels. Using a tetracycline-regulated system in human embryonic kidney 293 (HEK293) cells, O’Toole et al. also suggested that APOL1 overexpression drives variant-independent cytotoxicity [45]. In contrast, several other reports using HEK293 cells [24,27] or transduced human podocytes [22] showed APOL1 variant-dependent cytotoxicity. However, those studies used other methods for APOL1 induction, which might explain the difference with our findings.

In our study, as we were developing new podocyte cell lines, we chose to compare them with well-established human immortalised podocytes, which are the APOL1 G0/G0 control podocytes donated by Prof. Saleem [31]. However, these podocytes were obtained from a Caucasian donor, and this feature may have an influence on the APOL1 expression level in comparison with an African donor [8,46]. It has been reported previously that genetic variations are present in the human *APOL1* gene region containing multiple coding variants organised into distinct haplotypes, including at least seven relatively common non-risk haplotypes with different coding sequences [8]. The reference APOL1 haplotype that is extensively used in many studies is rarely found in Africans, but present in approximately 20–25% of the Caucasian and Asian populations [46]. Therefore, we propose that future experiments regarding APOL1 cell biology ideally should also include controls derived from African donors. 

Interestingly, the patient from whom we derived the APOL1 G2/G2 cell line was born prematurely due to pre-eclampsia and suffered post-neonatal asphyxia, which might have contributed to the decreased eGFR and mild proteinuria (Table 2). Although additional mutations could have contributed to the cellular phenotype, our DNA sequencing study was focused on the APOL1 risk variants and we did not perform whole-genome sequencing. 

In addition, treatment with poly(I:C) induces high overexpression of APOL1 in all podocyte genotypes, which might be too harsh for the cells, hiding the specific toxicity related to variant specific APOL1 overexpression. We suggest that the direct induction of APOL1 with interferon [22] might be an alternative to induce a weaker but more physiological upregulation of APOL1. 

Interestingly, our podocyte cell model showed an overall reduction in the autophagic flux in G2/G2 compared with G0/G0 podocytes in basal conditions, which was enhanced after treatment with poly(I:C). Several studies reported that overexpression of APOL1 led to altered autophagic cell death in different cell types [36,47]. Using TRE-APOL1 constructs in HEK293 cells, a lower degree of autophagic flux in cells transfected with a risk allele (as compared to G0-allele-expressing cells) was demonstrated. Further analysis confirmed that risk allele-transfected cells contained a greater amount of autophagic vacuoles, but fewer autolysosomes compared with cells transfected with the reference allele, indicating a blockage in autophagic flux [24]. The relevance of these findings was shown in human APOL1 HRG G1/G2 podocytes and is now confirmed by our G2/G2 model. In contrast with the aforementioned results, using an HEK293 cell model, O’Toole et al. examined autophagy in three clones of each genotype (G0, G1, G2) at 8 and 16 h after tetracycline induction and found that APOL1 overexpression did not affect autophagy at these two time points and that the autophagic flux was not altered by overexpression of APOL1-G0, G1 or G2 [45]. Again, it becomes clear that differences in cell types and protocols used for induction of APOL1 overexpression might underlie these conflicting results and warrant further study.

Various mechanisms underpinning glomerular pathologies converge on abnormal podocyte cytoskeleton organisation [38]. Differences in podocyte cytoskeleton and its consequences were the most striking findings in our study. It has been suggested that APOL1 RVs promoted podocyte dedifferentiation with decreased expression of podocyte-specific genes [48,49]. Interestingly, we observed an endogenous upregulation of synaptopodin in all clones of G2/G2 in comparison with G0/G0 and a consequent downregulation trend of this gene after treatment with poly(I:C) (Figure 2D). Synaptopodin is an actin-associated protein of differentiated podocytes. It plays a role in modulating actin-based shape and motility of podocyte foot processes. Thus, a reduced expression of synaptopodin leads to an alteration of the podocyte cytoskeleton. We analysed the actin filaments of G2/G2 podocytes in comparison with G0/G0 before and after APOL1 overexpression. Our results showed that podocyte cytoskeleton was differentially arranged, and actin filaments appeared to be disorganised in poly(I:C) induced APOL1 G2/G2 podocytes compared with the treated control cells. Furthermore, induced G2/G2 podocytes showed a lower number and distribution of adhesion sites than controls. These results suggest alteration of podocyte cytoskeleton driven by APOL1 upregulation.

Using a lentivirus transduced human podocyte cell model, Lan et al. reported that overexpression of APOL1-G1 and G2 dramatically increased lysosomal membrane permeability and the leakage of lysosomal components into the cytosol, which was accompanied by APOL1-induced F-actin degradation and necrotic cell death in podocytes [22]. As a possible mechanism of disorganised actin cytoskeleton in podocytes carrying RVs, Kumar et al. reported using transduced human podocyte cells that APOL1-miR193a preserves the integrity of actin cytoskeleton in differentiated podocytes through the stabilisation of the adherence complex, while disruption of APOL1-miR193a axis in podocytes expressing APOL1 risk alleles induces loss of this function. More specifically, their data showed that overexpression of APOL1 in G0 podocytes downregulated the expression of miR193a, while the expression of APOL1 in G1 and G2 podocytes was associated with upregulation of miR193a resulting in a disorganised actin cytoskeleton [48]. Further studies should be performed to elucidate the functional significance of these findings. Still, it is well accepted that abnormal podocyte cytoskeleton and adhesion sites result in proteinuria and glomerulosclerosis [38]. Indeed, in an assay to mimic the glomerular filtration in vitro under constant flux in dynamic conditions, our APOL1 G2/G2 podocytes treated with poly(I:C) showed increased permeability to albumin compared with baseline.

## 5. Conclusions

In conclusion, APOL1 HRG (G2/G2) podocyte cell lines generated from a human donor represent a robust and powerful tool to further study the mechanisms of APOL1-induced kidney injury.

## Figures and Tables

**Table 1:** Primer sequences used for gene expression analyses in quantitative PCR.

Target	Forward Primer	Reverse Primer
*CD2AP*	AGGCTGGTGGAGTGGAAC	CAGGAAGGTATAGGTGAAGTAGG
*Synaptopodin*	AGCCCAAGGTGACCCCGAAT	CCCTGTCACGAGGTGCTGGC
*Podocalyxin*	CTTGAGACACAGACACAGAG	CCGTATGCCGCACTTATC
*APOL1*	AATGAGGCCTGGAACGGAT	TCAACCGAGGAAACTCTTTCA
*β-actin*	AAGAGCTACGAGCTGCCTGA	GACTCCATGCCCAGGAAGG

**Table 2:** Baseline characteristics of participants carrying *APOL1* HRG.

Participant	Age (Years)	Gender	Country	Medical History	eGFR (mL/min/1.73 m^2^)	P/C Ratio mg/mg	*APOL1* Genotype
1	6	M	Ghana	Kidney injury post neonatal asphyxia	28	0.15	G2/G2
2	28	F	Cameroon	Normal	NA	NA	G1/G2
3	37	M	DRC	Hypertension	98	0.25	G1/G1
4	38	F	DRC	Normal	104	0.18	G1/G2
5	40	M	DRC	Normal	NA	NA	G1/G2
6	43	F	DRC	Sickle cell trait	112	0.12	G1/G2

eGFR: estimated glomerular filtration rate; P/C ratio: protein to creatinine ratio; NA: data not available; DRC: Democratic Republic of Congo; M: male; F: female.

## Data Availability

The datasets generated during and/or analysed during the current study are available from the corresponding author on reasonable request.

## Supplementary Materials

The following are available online at https://www.mdpi.com/article/10.3390/cells10081914/s1, Figure S1: Full length blots of LC3-II western-blot analysis.
