# Supplementary material for: Novel Human Podocyte Cell Model Carrying G2/G2 APOL1 High-Risk Genotype
Source: Cells. 2021 Jul 28;10(8):1914. doi: 10.3390/cells10081914 (PMC8391400; doi:10.3390/cells10081914)
Supplement: Supplementary file 1 [file cells-10-01914-s001.zip › cells-1268787-supplementary.pdf]

Supplementary Material

Novel Human Podocyte Cell Model Carrying G2/G2 APOL1 High-Risk Genotype

[§]**Pepe M. Ekulu**[1,2], [§]**Oyindamola C. Adebayo**[1,3], Jean-Paul Decuypere[1], Linda Bellucci[4], Mohamed A. Elmonem[5], Agathe B. Nkoy[1,2], Djalila Mekahli[1,6], Benedetta Bussolati[4], Lambertus P. van den Heuvel[1,7], **#Fanny O. Arcolino**[1]*, **#Elena N. Levtchenko**[1,6]

1. Department of Development and Regeneration, Katholieke Universiteit Leuven, 3000 Leuven, Belgium; christiana.adebayo@kuleuven.be (O.C.A.); fanny.oliveiraarcolino@kuleuven.be (F.O.A); jeanpaul.decuypere@kuleuven.vib.be (J.P.D.); agathe.nkoybikupe@student.kuleuven.be (A.B.N.); bert.vandenheuvel@kuleuven.be (L.P.v.d.H.)

2. Division of Nephrology, Department of Paediatrics, University Hospital of Kinshasa, Faculty of Medicine, University of Kinshasa, Democratic Republic of Congo); drmfutu@yahoo.fr (P.M.E.)

3. Centre for Molecular and Vascular Biology, Department of Cardiovascular Sciences, Katholieke Universiteit Leuven, 3000 Leuven, Belgium.

4. Department of Molecular Biotechnology and Health Sciences, University of Turin, 10124 Turin, Italy; linda.bellucci@unito.it (L.B.); benedetta.bussolati@unito.it (B.B.)

5. Department of Clinical and Chemical Pathology, Faculty of Medicine, Cairo University, 11628 Cairo, Egypt); mohamed.abdelmonem@kasralainy.edu.eg (M.A.E)

6. Department of Paediatrics, Division of Nephrology, University Hospitals Leuven, 3000 Leuven, Belgium; djalila.mekahli@uzleuven.be (D.M.); elena.levtchenko@uzleuven.be (E.N.L.).

7. Department of Paediatric Nephrology, Radboud University Medical Centre, 6500 Nijmegen, the Netherlands

§ Shared-first authors

# Shared-last authors

*** Corresponding author:**

Dr. Fanny Oliveira Arcolino; Email: fanny.oliveiraarcolino@kuleuven.be

Address: Department of Development and Regeneration, Katholieke Universiteit Leuven, Herestraat 49, Box 817, 3000 Leuven, Belgium.

Telephone: +32-16372647

Supplementary figure

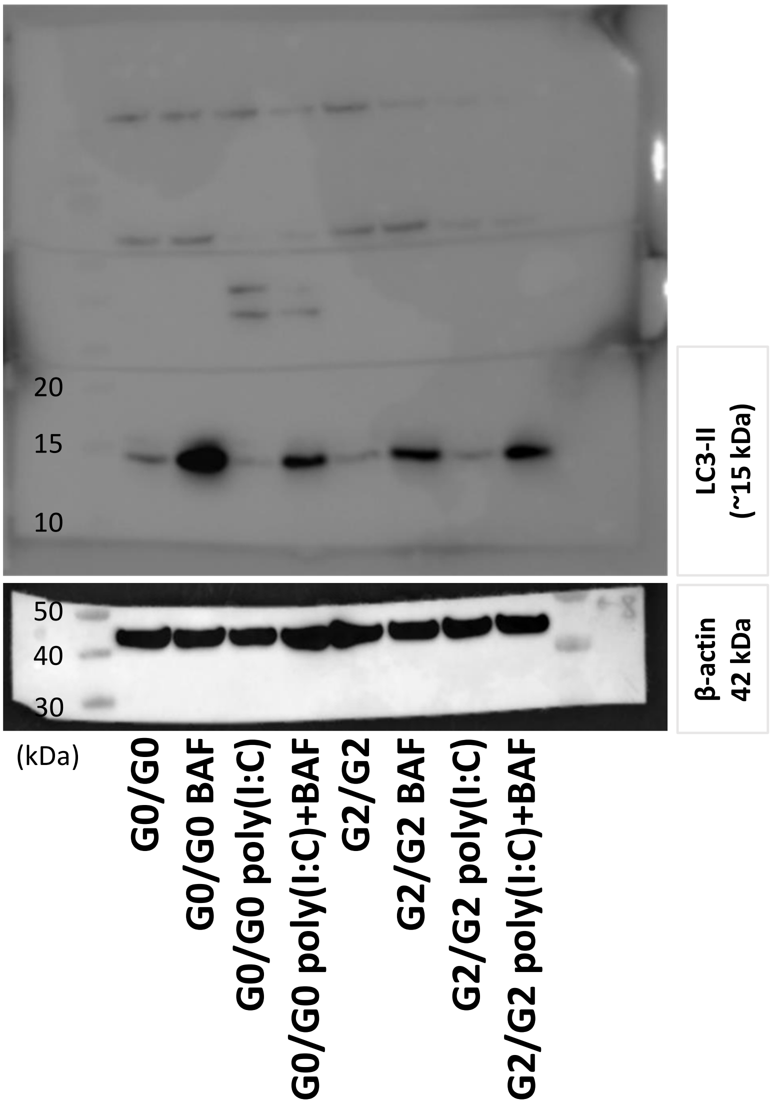

Figure S1. Full length blot of LC3-II western-blot analysis. LC3-II western-blot analysis of G0/G0 and G2/G2 podocyte cell lines in: basal level (G0/G0; G2/G2); after treatment with bafilomycin A (Baf, 100 nM) to induce an autophagic block; after 24-hour incubation with poly(I:C) (50 ng/ml) to induce upregulation of APOL1; and combined induction with poly(I:C) and treatment with bafilomycin A (poly I:C+Baf).
